# Machine Learning for Clinical Decision-Making: Challenges and Opportunities in Cardiovascular Imaging

**DOI:** 10.3389/fcvm.2021.765693

**Published:** 2022-01-04

**Authors:** Sergio Sanchez-Martinez, Oscar Camara, Gemma Piella, Maja Cikes, Miguel Ángel González-Ballester, Marius Miron, Alfredo Vellido, Emilia Gómez, Alan G. Fraser, Bart Bijnens

**Affiliations:** ^1^August Pi i Sunyer Biomedical Research Institute (IDIBAPS), Barcelona, Spain; ^2^Department of Information and Communication Technologies, University Pompeu Fabra, Barcelona, Spain; ^3^Department of Cardiovascular Diseases, University of Zagreb School of Medicine, University Hospital Centre Zagreb, Zagreb, Croatia; ^4^ICREA, Barcelona, Spain; ^5^Joint Research Centre, European Commission, Seville, Spain; ^6^Computer Science Department, Intelligent Data Science and Artificial Intelligence (IDEAI-UPC) Research Center, Universitat Politècnica de Catalunya, Barcelona, Spain; ^7^School of Medicine, Cardiff University, Cardiff, United Kingdom; ^8^Department of Cardiovascular Sciences, KU Leuven, Leuven, Belgium

**Keywords:** artificial intelligence, machine learning, deep learning, clinical decision making, cardiovascular imaging, diagnosis, prediction

## Abstract

The use of machine learning (ML) approaches to target clinical problems is called to revolutionize clinical decision-making in cardiology. The success of these tools is dependent on the understanding of the intrinsic processes being used during the conventional pathway by which clinicians make decisions. In a parallelism with this pathway, ML can have an impact at four levels: for data acquisition, predominantly by extracting standardized, high-quality information with the smallest possible learning curve; for feature extraction, by discharging healthcare practitioners from performing tedious measurements on raw data; for interpretation, by digesting complex, heterogeneous data in order to augment the understanding of the patient status; and for decision support, by leveraging the previous steps to predict clinical outcomes, response to treatment or to recommend a specific intervention. This paper discusses the state-of-the-art, as well as the current clinical status and challenges associated with the two later tasks of interpretation and decision support, together with the challenges related to the learning process, the auditability/traceability, the system infrastructure and the integration within clinical processes in cardiovascular imaging.

## Introduction

Artificial intelligence (AI) systems are programmed to achieve complex tasks by perceiving their environment through data acquisition, interpreting the collected data and deciding the best action(s) to take to achieve a given goal. As a broad scientific discipline, AI includes several approaches and techniques, such as machine learning, machine reasoning, and robotics (1). Machine learning (ML) is the subfield of AI that focuses on the development of algorithms that allow computers to automatically discover patterns in the data and improve with experience, without being given a set of explicit instructions. Among ML techniques, Deep Learning (DL) is the subfield concerned with algorithms inspired by the structure and function of the brain called artificial neural networks. Unlike other ML techniques, DL bypasses the need of using hand-crafted features as input, automatically figuring out the data features that are important for solving complex problems. This is the main reason why DL stands out as the current state-of-the-art in virtually all medical imaging related tasks.

In the particular case of clinical decision-making in cardiology, ML methods would perceive an individual by collecting and interpreting his/her clinical data and would reason on them to suggest actions to maintain or improve that individual's cardiovascular health. This mimics the clinician's approach when examining and treating a sick patient, or when suggesting preventive actions to avoid illness. Therefore, in order to assess the challenges and opportunities of ML systems for clinical decision-making in cardiology, an in-depth understanding of this process, when performed by cardiologists, is paramount.

**Figure 1:**
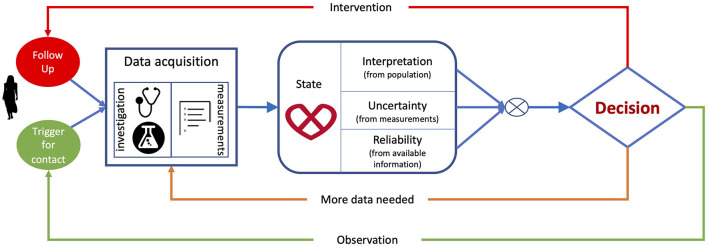
Clinical decision-making flowchart, from data acquisition and extraction, to patient's status interpretation and associated decision.

Figure 1 summarises a typical paradigm for clinical decision-making. It starts by data acquisition, including the clinical history of the patient, demographics, physiological measurements, electrocardiogram, imaging and laboratory tests, and the relevant indices collected from these data. Next, clinicians construct and interpret the state of the patient by comparison with population-based information learned during their education or daily practice, or information derived from guidelines. This interpretation is based on reasoning on the data using the human innate capability of contextualizing information through pattern recognition. Furthermore, clinicians assess the uncertainty associated with measurements and the completeness of the available information to estimate how much they can rely on the data. Finally, they consider the knowledge from the (natural as well as treated) expected evolution of populations related to the patient's status to make decisions. The resulting actions can be to either collect more data to minimize the uncertainty associated with the decision, to make an intervention (drug/device therapy, surgery, etc.) to improve the patient's outcomes, or to send the patient home (whether or not with planned observation follow-up).

In the era of evidence-based, personalised medicine (2), millions of individuals are carefully examined, which results in a deluge of complex, heterogeneous data. The use of algorithmic approaches to digest these data and augment clinical decision-making is now feasible due to the ever-increasing computing power, and the latest advances in the ML field (3). Indeed, big data leveraged by ML can provide well-curated information to clinicians so they can make better informed diagnoses and treatment recommendations, while also estimating probabilities and costs for the possible outcomes. ML-augmented decisions made by clinicians have the potential to improve outcomes, lower costs of care, and increase patient and family satisfaction.

**Figure 2:**
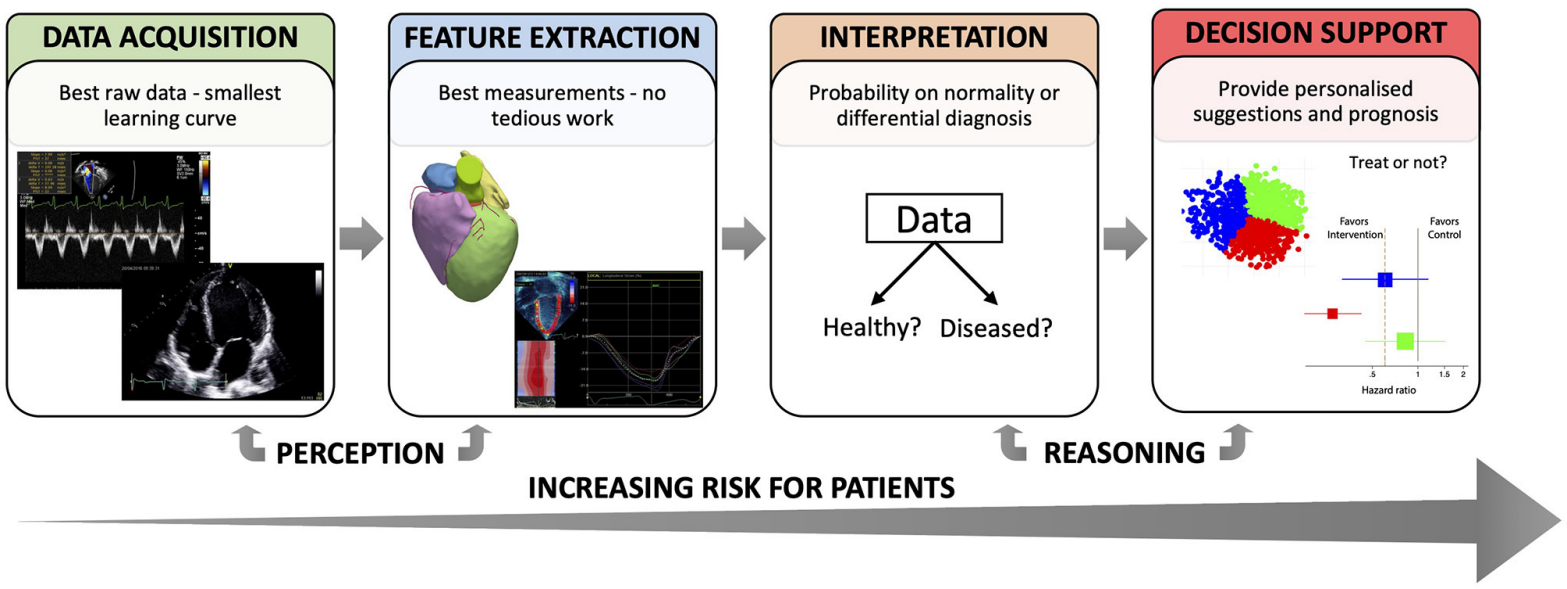
Different tasks where ML can support clinical decision-making.

ML analyses have, to date, demonstrated human-like performance in low-level tasks where pattern recognition or perception play a fundamental role. Some examples are **data acquisition**, standardization and classification (4, 5), and **feature extraction** (6, 7). For higher-level tasks involving reasoning, such as patient's **status interpretation** and **decision support**, ML allows for the integration of complex, heterogenous data in the decision-making process, but these are still immature and need substantial validation (8). In parallel to Figure 1, which illustrated the process of making clinical decisions, Figure 2 describes the tasks involved in this process according to how ML could contribute, and highlights how the risks to a patient from erroneous conclusions increase with each step.

There exist other review papers that cover the topic of AI in cardiovascular imaging from a broader perspective (9), or that highlight the synergy between machine learning and mechanistic models that enable the creation of a “digital twin” in pursuit of precision cardiology (10). Complementarily, this paper focuses on ML as a subfield of AI and on clinical decision-making as an essential part of cardiovascular medicine. Although cardiovascular imaging only constitutes a limited portion of the data spectrum in cardiology, we emphasize the imaging field in our literature review given that it is one of the areas to which ML has contributed the most (11). In the following, we discuss the higher-level tasks related to clinical decision-making that involve reasoning on clinical data, namely interpretation, and decision support. For each of these tasks, we review the ML state-of-the-art (indicating whether the implementation was based on DL or other ML technique), comment on the current penetration of ML tools into clinical practice (see *Clinical status* subsections), and elaborate on the current challenges that limit their implementation in clinical practice. Finally, we discuss the general challenges that may appear when tackling any clinical problem with ML approaches.

This paper addresses potential questions arising from data scientists, industrial partners and funding institutions, helping them understand clinical decision-making in cardiology and identify potential niches for their solutions to be helpful. At the same time, the paper aims at informing cardiologists about which ML tools could target their problems and what are their current limitations.

## Status Interpretation—Comparison to Population

Let us assume that the clinical data of a patient have been properly acquired and relevant features are readily available. The next stage in the decision process consists in interpreting his/her status by comparison to populations. This comparison requires data normalization. When complex data are involved, such as cardiac images, the traditional approach to normalization is to build a statistical atlas–a reference model that captures the variability associated to a population (12). To build the atlas, the training data must be transformed into a common spatio(-temporal) framework, which can be achieved by registration. In this sense, registration appears as a crucial step for status interpretation toward diagnosis, and deep learning has emerged as a suitable tool to register 3D cardiac volumes (13), 3D pre-operative cardiac models to 2D intraoperative x-ray fluoroscopy to facilitate image-guided interventions (14), or cardiac MRI sequences (15).

The ML interpretation of the state of the patient can augment the diagnosis made by clinicians. Indeed, a recently published meta-analysis highlighted the promising potential of ML and DL models to predict conditions such as coronary artery disease, heart failure, stroke, and cardiac arrhythmias using data derived from routinely used imaging techniques and ECG (16). Based on imaging, ensemble ML models, which group the prediction of different weak learners, have demonstrated higher accuracy than expert readers for the diagnosis of obstructive coronary artery disease (17); a DL model automated the diagnosis of acute ischemic infarction using CT studies (18); and another DL model achieved 92.3% accuracy for left ventricular hypertrophy classification analysing echocardiographic images (19). Different ML models have also operated on electronic health records (EHR) for triaging of low-risk vs. high-risk cardiovascular patients, grading findings as requiring non-urgent, urgent or critical attention, as a strategy to improve efficiency and allocation of the finite resources available in the emergency department (20). Lastly, a ML ensemble model combined clinical data, quantitative stenosis, and plaque metrics from CT angiography to effectively detect lesion-specific ischemia (21).

Another data-driven example of status interpretation is unsupervised machine learning for dimensionality reduction; a label-agnostic approach that orders individuals according to their similarity, i.e., those with a similar clinical presentation are grouped together, whereas those showing distinct pathophysiological features are positioned far apart (22). This allows identifying different levels of abnormality, or assessing the effect of therapies and interventions, as these are aimed to restore an individual toward increased “normality.” An implementation of unsupervised dimensionality reduction provided useful insight into treatment response in large patient populations (23), and quantified patient changes after an intervention using temporally dynamic data (24).

### Clinical Status

ML approaches for interpreting a patient's status enhance discovery in massive databases by offering the possibility to identify similar cases, build normality statistics, and spot outliers. Whether these approaches are intended for diagnosis or risk assessment, they could contribute to deliver better healthcare.

Unfortunately, many current ML applications for interpreting clinical data present a technically sound contribution, but do not address real clinical needs, and they often focus on binary classification of normal vs. abnormal (19), which strongly limits their use in routine clinical practice. Furthermore, studies showing impact on hard clinical endpoints rather than on surrogate measures are still needed. The way forward is through further integration of technical and clinical contributions, and through the elaboration of consensus recommendations on how to tackle a clinical necessity using ML.

## Decision-Making (Prediction)

Based on the interpretation of the patient's status, clinicians should decide on whether: (1) observe the patient and wait until an event triggers the need for a decision; (2) collect more data to improve the odds of making the right decision; or (3) perform an intervention and monitor the outcome (see Figure 1). Machine learning methods can help clinicians to decide which pathway to follow (25), in a way that is cost-effective (26).

Several studies have assessed the predictive power of ML techniques based on imaging. An echocardiography-based DL model was shown to improve the prediction of in-hospital mortality among coronary heart disease and heart failure patients as compared to traditionally used prediction models (27). An ensemble ML approach interrogating SPECT myocardial perfusion studies demonstrated superior performance at predicting early revascularization in patients with suspected coronary artery disease as compared to an experienced reader (28), or in combination with clinical and ECG data outperformed the reading physicians at predicting the occurrence of major adverse cardiovascular events (29). Lastly, a DL implementation fed with CT scans from asymptomatic as well as stable and acute chest pain cohorts demonstrated the added clinical value of automated systems to predict cardiovascular events (30). Leaving imaging aside, deep learning based on clinical, laboratory and demographic data, ECG parameters, and cardiopulmonary exercise testing estimated prognosis and guided therapy in a large population of adults with congenital heart disease (31).

The interplay between different a priori non-related imaging tests has recently been discovered by DL through the identification of previously unnoticed associations. For example, breast arterial calcifications and the likelihood of patients at a high cardiovascular risk was sorted out using a DL model that operated with mammograms (32). Similarly, the power of ML in combination with the non-invasiveness of retinal scanning has been used to predict abnormalities in the macrovasculature based on the microvascular features of the eye. One such example is the DL model that predicted cardiovascular risk factors using retinal fundus photographs, thus allowing for an easier and cost-effective cardiovascular risk stratification (33), or the DL implementation that inferred coronary artery calcium (CAC) scores from retinal photographs, which turned out to be as accurate as CT scan-measured CAC in predicting cardiovascular events (34).

### Clinical Status

The few examples of FDA-cleared cutting-edge ML applications to cardiovascular imaging that are thus suitable for routine use, focus on the low-level tasks of data acquisition and feature extraction, both in cardiac MRI (35) and echocardiography (36, 37), although the latter contribution did actually prove useful to predict a poor prognosis in acute COVID-19 patients based on DL-enabled automated quantification of echocardiographic images. However, the use of these ML applications for prediction and decision-making is still in its early days, as most models are still incapable of making predictions at the individual level (8, 38). More effort is needed toward integration in a clinical environment, interpretability, and validation if we want to see these models embedded in routine patient care.

## Challenges Common to Status Interpretation & Decision-Making

Applications concerning patient's status interpretation and decision-making, which entails learning what is the risk associated with each possible clinical decision, imply a much higher risk as compared to the low-level tasks of data acquisition and feature extraction, since decisions derived from them could harm patients. Accordingly, ML outcomes need to be intuitively interpretable by the cardiologist and validated in a much more exhaustive way (as required by medical device regulators; e.g., class IIa or IIb routes to commercialization), ultimately with the launch of randomized prospective trials.

One of the main challenges for ML approaches to status interpretation lies in the extraction of meaningful concepts from raw data. This challenge entails many others, related to the data themselves. The first concerns the reliability and representativeness of training and outcome data. If representative, ML models need to find a reliable metric to compare heterogeneous data, which is not trivial. Furthermore, for a successful interpretation, data collection protocols should be designed to cover gender-, ethnicity- and age-related changes, and capture the rare outliers (39). On top of this, ML systems should be designed to consider longitudinal data, as to assess a patient over time, e.g., during a stress protocol (40) or disease progression (41). Finally, ML models are trained on three different kinds of data; ranging from higher to lower quality and completeness: (1) randomized clinical trials, (2) cohorts, and (3) clinical routine real-world data. The exchange of knowledge throughout these collections of data is challenging, since what was learned from highly curated data (e.g., randomized clinical trials) may not generalize to routinely collected data.

Another crucial problem associated to currently available data is bias, i.e., when the training sample is not representative of the population of interest (see section “General challenges” for more details). Accordingly, caution is needed when testing a trained model in new clinical centres. As ML users can attest, there will always be a trade-off between improving the system performance locally and having systems that generalize well (42). Automation bias, defined as the human tendency to accept a computer-generated solution without searching for contradictory information (43), may also affect clinical interpretation and decision-making. As shown by Goddard et al. (44), when the ML solution is reliable it augments human performance, but when the solution is incorrect human errors increase. Thus, who is to blame if a diagnostic algorithm fails? The further we move along the clinical decision-making flowchart (Figure 2), the more ethical and legal barriers the ML practitioner/company faces. To mitigate some of these issues, the training data should be accessible, and the learning systems equipped with tools that allow reconstructing the reasoning behind the decision taken.

Table 1 organizes the ideas discussed for status interpretation and decision-making in the form of a SWOT analysis.

**Table 1:** SWOT analysis—status interpretation and decision-making.

Strengths	Weaknesses
• Allow objective and thorough comparison to populations • Allow the integration of complex, heterogenous features • May enhance the prediction of clinical outcomes, or the prediction of response to a given treatment or intervention.	• Need well-curated, representative databases for training • Affected by data reliability, representativeness, and bias • Need to extract meaningful, interpretable concepts • Need thorough validation–prospective trials • Need to integrate longitudinal data • Ensure transference of knowledge across populations • Need to prove clinical benefit • Need to be integrated within clinical systems • Need to prove cost-effectiveness
Opportunities	Threats
• Stimulate the man/machine collaboration • Reach diagnosis in a shorter time • Separate ambiguous cases that deserve more attention from clear cases–triaging • Help in the organization of healthcare—diagnosis, risk assessment and urgency assessment • Lower cost of healthcare by suggesting cost-effective decisions	• Harm patients if wrong decisions are taken—high-risk • Disappoint users, especially after all the striking news on ML failures • Affect human decisions in a negative way—automation bias • Make decisions for the average patient, not at the individual level

## General Challenges

We have previously described the specific challenges that may arise when ML models are given the tasks of interpreting the patient's status or making predictions to guide the clinical decision. In the following, we discuss the general challenges that may appear when tackling any clinical problem with ML approaches. These are divided into different sections, depending on whether they relate to the learning itself, the auditability/traceability aspects, the system/infrastructure, or the integration within clinical processes.

### Learning

#### (Non-standardized) Data

Medical data are normally kept in many separate systems, which hampers accessibility and makes comparisons at a population level nearly impossible. Electronic health records mostly contain unstructured data, and so they are underutilized by care providers and clinical researchers. Machine learning systems can help organize and standardize information, or can be designed to directly integrate unstructured complex data for high-throughput phenotyping to identify patient cohorts (45).

#### Bias and Confounding

As discussed in the previous section, bias is another risk that arises with the use of ML. Indeed, a recent review of cardiovascular risk prediction models revealed potential problems in the generalizability of multicentre studies that often show a wide variation in reporting, and thus these models may be biased toward the methods of care routinely used in the interrogated centres (46). For example, in the case of cardiac MRI, protocols are not standardised, varying by institution and machine vendor (47). This bias may amplify the gap in health outcomes between the dominant social group, whose data are used to train algorithms, and the minorities (8). Luckily enough, there are studies that make sure that all minorities are represented in the training data (12, 39), but this should be the rule, not the exception. Another challenge when applying ML models that are designed to recognise patterns lies in the tendency of these to overfit the dataset because they fail at distinguishing a true contributing factor to the clinical outcome from noise (48).

ML solutions can similarly inherit human-like biases (49), such as the model whose recommendation of care after a heart attack was unequal among sex groups (50), as a consequence of a biased training dataset. This bias may also appear in ML-powered echocardiography, where studies are dependent on both the operator performing the study and the interpreter analysing it (47). This bias occurs because we ask ML solutions to predict which decisions the humans profiled in the training data would have made, thus we should not expect the ML method to be fair. The effect of human cognitive biases in ML algorithms have already been addressed and different “debiasing” techniques have been proposed (51), but this is a topic that certainly needs more attention.

Similar to bias, the learning process can be undermined by confounding, i.e., the finding of a spurious association between the input data and the outcome under study. Such is the case of the deep learning model that attempted at predicting ischaemia by looking at ECG records, but rather learnt at detecting the background electrical activity noise present in the ischaemic ECG examples, which was not present in the control cases (52). Unsupervised learning can be beneficial to avoid confounding, as it does not force the output of the model to match a given label but rather finds natural associations within the data (53). Similarly, randomization of experiments is highly recommended to avoid confounding (54).

#### Validation and Continuous Improvement

Even if an algorithm proves to outperform humans in prediction tasks, systematic debugging, audit and extensive validation should be mandatory. For ML algorithms to be deployed in hospitals, they must improve patient as well as financial outcomes (8). Validation should be through multi-centre randomized prospective trials, to assess whether models trained at one site can be applied elsewhere. Examples of prospective ML trials assessed in a “real world” clinical environment are scarce–only 6% of 516 surveyed studies performed external validation, according to (55). Among these rare examples, finding prospective validation studies to prove the suitability of ML-enabled applications in cardiovascular imaging is even rarer. In (56), a prospective study concluded that a ML model that integrates clinical and quantitative imaging-based features outperforms the prediction of myocardial infarction and death as compared to standard clinical risk assessment. Attia et al. (57) conducted a prospective study to validate a DL algorithm that detected left ventricular systolic dysfunction. Another pivotal prospective multicentre trial was launched to demonstrate the feasibility of a ML-powered image guided acquisition software that helps novices to perform transthoracic echocardiography studies (58). Lastly, a validation study was performed to prove the feasibility of using DL to automatically segment and quantify the ventricular volumes in cardiac MRI (35).

One of the greatest benefits of ML models resides in their ability to improve their performance as more data become available. However, this might be challenging particularly for neural networks, which are prone to “catastrophic forgetting”—to abruptly forget previously learned information upon learning from new data. Furthermore, re-training on the whole database is time and resource consuming. To solve these problems, federated learning, a novel de-centralized computational architecture where machines run models locally to improve them with a single user‘s data (59), could be helpful. Given the evolving nature of ML models, medical device providers are obliged to periodically monitor the performance of their programs, using a continuous validation paradigm (60).

### Auditability/Traceability

#### Interpretability vs. Explainability

Interpretability is understood as the ability to explain or to present in understandable terms to a human (61). In a strictly-regulated field such as cardiovascular medicine, the lack of interpretability of ML models is one of the main limitations hindering adoption (62). Indeed, from the example of predicting ischaemia by looking at ECG records discussed above (52), it is evident that the non-intelligible use of ML outputs can lead to controversial results and therefore translation to clinical practice should be done cautiously. Unfortunately, many ML implementations available do not comply with the European General Data Protection Regulation (GDPR), which compels ML providers to reveal the information and logic involved in each decision (63).

When reasoning on the data to make decisions, the human brain can follow two approaches (64): the *fast/intuitive* (Type 1) vs. the *slow/reasoned* (Type 2) one. Type 1 is almost instantaneous and based on the human ability to apply heuristics to identify patterns from raw information. However, it is prone to error and bias, as it can lead to an incomplete *perception* of the patient due to low quality or lack of relevant data (65, 66). In contrast, Type 2 is deductive, deliberate, and demands a greater intellectual, time and cost investment, but often turns out to be more accurate.

The above is highly relevant for both “traditional” and ML-based clinical decision-making, as ML systems ultimately mimic different aspects of human reasoning and can lead to the same errors. For the sake of explaining ML decisions, researchers provide attention maps (67), reveal which data the model “looked at” for each individual decision (68), provide estimates of feature importance (69), or explain the local behaviour of complex models by changing the input and evaluating the impact on the prediction (70). However, caution is needed with this entire research trend, as explainability is not a synonym of interpretability (71). Explainable models tend to reach conclusions by fast/intuitive black-box reasoning (Type 1, see also causal vs. predictive learning in the following subsection), while interpretable models demand a slow/reasoned (Type 2) approach throughout the entire learning path. In this sense, explainable models that follow a fast/intuitive reasoning might incur more diagnostic errors than interpretable models, which follow an analytical reasoning (64). The research field that focuses on enhancing models' interpretability is still in its infancy. Generative synthesis (69), which uses ML to generate a simplified version of a neural network, or mathematical attempts to explain the inner working of neural networks (72) could provide key insight into why and how a network behaves the way it does, thus unravelling the black-box enigma (73).

For ML models to be applied in clinical decision-making, they cannot merely be interpretable, but they also must be credible. A credible model is an interpretable model that: (1) provides arguments for its predictions that are, at least in part, in-line with domain knowledge; (2) is at least as good as previous standards in terms of predictive performance (74). For ML models to achieve credibility, the medical expert must be included in the interpretation loop (75).

Together with models, we should also develop strategies to objectively evaluate their interpretability. This can be done by assessing fairness; privacy of data; generalizability; causality, to prevent spurious correlations; or trust, to make sure the model is right for the right reasons (61). Depending on the application, the interpretability needs might be different. For cardiology applications in particular, ML for status interpretation and decision-making should be equipped with the most-advanced interpretability tools.

#### Causal ML Rather Than Predictive ML

Predictive ML based on correlations of input data and outcomes may not be enough to truly impact the healthcare system. Indeed, this form of learning can be misleading if important causal variables are not analysed. For auditability reasons, we should probably shift toward finding the root causes of *why* that decision was made, and interpreting the process followed by the algorithm to reach that (diagnostic) decision, i.e., *how* the diagnosis was made. These two questions are addressed by causal ML, a powerful type of analysis aimed at inferring the mechanisms of the system producing the output data. In practice, causal models provide detailed maps of interaction between variables, so the users can simulate cause and effect of future actions (76).

### System-Related

#### Security

Machine learning raises a handful of data security and privacy issues, as DL models require enormous datasets for training purposes. The most secure way to transfer data between healthcare organizations is still unclear, and stakeholders no longer underestimate the hazards of a high-profile data leakage. Hacking is even more harmful, as hackers could manipulate a decision-making model to damage people at a large scale.

The European GDPR compels to adopt security measures against hacking and data breaches. A potential solution that has been largely discussed is Blockchain, a technology that enables data exchange systems that are cryptographically secured and irrevocable, by providing a public and immutable log of transactions and “smart contracts” to regulate data access. The downsides of Blockchain's technology are that it is slow, costly to maintain, and hard to scale (77). As an alternative, federated learning could guarantee the security of patient data (see “Validation and continuous improvement subsection”), as this model-training paradigm allows updating a learning model locally without sharing individual information with a central system (78).

#### Regulatory

The use of ML for clinical decision-making unavoidably brings legal challenges regarding medical negligence derived from learning failures. When such negligence arises, the legal system needs to provide guidance on what entity holds liability, for which recommendations have been developed (79). Furthermore, the evolving nature of ML models poses a unique challenge to regulatory agencies, and the best way to evaluate updates remains unclear (60). Policymakers should generate specific criteria for demonstrating non-inferiority of algorithms compared to existing standards, specially emphasizing the quality of the training data and the validation process (80). Regulatory bodies must also ensure that algorithms are used properly and for people's welfare. In summary, for ML technology to be adopted by cardiology departments within the next years many legal aspects still need to be addressed, and decision- and policy-makers should join efforts toward this end.

### Integration (Man/Machine Coexistence)

The scenario of ML tools replacing humans in clinical medicine is highly unlikely (81). Besides the formidable challenges for ML solutions discussed above (8), cardiologists will still be needed to interact with the patients and perform physical examinations, navigate diagnostic procedures, integrate and adapt ML solutions according to the changing stages of disease or patient's preferences, inform the patient's family about therapy options, or console them if the disease stage is very advanced.

Accordingly, instead of a human-machine competition, we should rather think of a cooperation paradigm, where ML is used to augment human intelligence–targeting repetitive sub-tasks to assist physicians to reach a more informed decision, more efficiently. Indeed, ML and humans possess complementary skills: ML stands out at pattern recognition on massive amounts of data, whereas people are far better at understanding the context, abstracting knowledge from their experience, and transferring it across domains. Human-in-the-loop approaches facilitate cooperation by enabling users to interact with ML models without requiring in-depth technical knowledge. However, understanding where ML models can be used and at which level is crucial to avoid preventable errors attributed to automation bias (43). Examples of human-machine collaboration already exist. Indeed, a ML algorithm cleared by the FDA improved the diagnosis of wrist fractures when clinicians used it, as compared to clinicians alone (82). In diabetic retinopathy diagnosis (83), model assistance increased the accuracy of retina specialists above that of the unassisted reader or model alone. In cardiovascular imaging, most examples of human-machine collaboration thus far focus on segmentation, and detection-classification of imaging planes (84).

In light of this, the current clinical workflow could be rethought: the ML system would propose a diagnosis, the human operator then revises the data on which the conclusions are drawn, informing the system of potential measurement errors or confounders, and finally accepts or rejects the diagnosis. Thus, the human operator preserves the overall control, while machines perform measurements and integrate and compare data at request (75). Ultimately, this human-machine symbiosis will be beneficial to release physicians from low-level tasks such as cardiac measurements, data preparation, and standardization, to give them more time on higher-level tasks such as patient care and clinical decision-making (85).

### ML Applied to Real Clinical Data

In human decision-making, a clinician would explore all available data and compare them to patients they have seen before or were trained to recognize. Once an individual is put into context with regards to expected normality and typical cases, previous knowledge on treatment effect is used to manage this individual patient. This ‘eminence-based’ approach is only within reach of very experienced clinicians. For standardization, many professional organizations provide diagnostic guidelines based on data from large cohorts or clinical trials (86–88). Although guidelines have significantly contributed to improve medical care, they do not consider the full data available. In this sense, the use of ML seems amply justified.

Most ML models are trained with data collected following strict input criteria and well-defined protocols used in randomized clinical trials (89, 90), but routinely collected data is often much noisier, heterogeneous and incomplete. ML techniques need to deal with incompleteness, either by performing imputation or by adopting formulations that explicitly consider that the data can be incomplete. Furthermore, patients often lie outside the narrow selection criteria of cardiology trials (including co-morbidities, ethnicity, gender, age, lifestyle, etc.), may have been differently treated before the investigation, may present at a different stage of disease, and most importantly, may undergo different decision pathways during the study. On top of this, obtaining a hard outcome to train an algorithm is often difficult, e.g., to register death, the study would need to be conducted until everybody dies, which is unfeasible both for time and economic constraints. Even if registered, often the outcome is scarce, and when appearing, the reason for experiencing it may be different among patients (91).

All these aspects make it extremely challenging to associate input descriptors to outcomes using supervised predictive ML/DL techniques, which may fail to understand the context from which data have been drawn, and thus yield unwanted results that might harm patients. A more promising approach could be based on unsupervised dimensionality reduction, a label-agnostic approach where input descriptors are used to position individuals according to their similarity and combined with previous knowledge this similarity can be used to infer diagnosis or to predict treatment response (23).

## Future Perspectives

The foreseeable application of ML in the short to mid-term is to perform specific and well-defined tasks relating to data acquisition, predominantly by extracting standardized, high-quality information with the smallest possible learning curve. In this sense, DL solutions already help extracting information with minimal or even without the need of human intervention (8, 92), or aid selecting the images that are good enough for subsequent clinical interrogation (93). Another evident application of ML that will soon be ubiquitous in clinical practice is that of image analysis, which will discharge cardiologists from monotonous activities related to feature extraction from images (94), thus freeing them up to dedicate more time to higher-level tasks involving interpretation, patient care, and decision-making.

For the topics covered in this paper, i.e., the higher-level tasks involving reasoning, such as patient's status interpretation and decision support, ML applications are still immature and need substantial validation. A modest number of ongoing clinical trials have been conceived to tackle these drawbacks. One such example is the current investigation aiming at validating a DL model that diagnoses different arrhythmias (AF, supraventricular tachycardia, AV-block, asystole, ventricular tachycardia and ventricular fibrillation) on 12-lead ECGs and single-lead Holter monitoring registered in 25,458 participants (95). Another example is the clinical study that will interrogate stress echocardiography scans with ML models to discriminate normal hearts from those at risk of a heart attack in a prospective cohort of 1,250 participants (96). Considering the above, we do not expect to see a vast penetration of ML-enabled applications for patient's status interpretation and decision support in clinical practice in the foreseeable future.

Whatever the application, the penetration of ML models into routine practice will be subject to their seamless integration into the clinical decision pathway used by cardiologists. Furthermore, we consider that the upcoming policies for ML research in healthcare should address the challenges described in the previous section, which can only be achieved by multidisciplinary teams. On the algorithmic side, more attention should be dedicated to dealing with longitudinal data, and how to relate the ML conclusions with pathophysiological knowledge. Data integration and what is the best approach for dealing with incomplete data and outliers should be also surveyed. On the validation side, generalization performance should be systematically reported, and uncertainty quantification methods should be developed to establish trust in the (predictive) models. Finally, the practical considerations that will affect adoption of the ML technology, such as how ML software should be integrated with the archiving and communication system of the hospital or how it would be paid for by facilities, should be explored. For these, a clear demonstration of the cost-effectiveness of ML technology in healthcare systems and its positive impact on patients' outcomes is needed.

## Conclusion

ML algorithms allow computers to automatically discover patterns in the data and improve with experience. Together with the enormous computational capacities of modern servers and the overwhelming amount of data resulting from the digitalization of healthcare systems, these algorithms open the door for a paradigm shift in clinical decision-making in cardiology. However, their seamless integration is dependent on the understanding of the intrinsic processes being used during the conventional pathway by which clinicians make decisions, which in turn helps identifying the areas where certain types of ML models can be most beneficial. If the obstacles and pitfalls that have been covered in this paper can be addressed satisfactorily, then ML might indeed revolutionize many aspects of healthcare, including cardiovascular medicine. For the promise to be fulfilled, engineers and clinicians will need to engage jointly in intensive development and validation of specific ML-enabled clinical applications.

## Author Contributions

SS-M participated in the conception and design of the review, drafted the work, approved the final version and agreed on the accuracy and integrity of the work. OC, GP, MC, MG-B, MM, AV, EG, and AF helped drafting the review, revised it critically for important intellectual content, approved the final version, and agreed on the accuracy and integrity of the work. BB participated in the conception and design of the review, drafted the work, approved the final version, and agreed on the accuracy and integrity of the work. All authors contributed to the article and approved the submitted version.

## Funding

This study was supported by the Spanish Ministry of Economy and Competitiveness (María de Maeztu Programme for R&D [MDM-2015-0502], Madrid, Spain) and by the Fundació La Marató de TV3 (n°20154031, Barcelona, Spain). The work of SS-M was supported by IDIBAPS and by the HUMAINT project of the Joint Research Centre of the European Commission. AV's contribution is funded by Spanish research project PID2019-104551RB-I00.

## Conflict of Interest

The authors declare that the research was conducted in the absence of any commercial or financial relationships that could be construed as a potential conflict of interest.

## Publisher's Note

All claims expressed in this article are solely those of the authors and do not necessarily represent those of their affiliated organizations, or those of the publisher, the editors and the reviewers. Any product that may be evaluated in this article, or claim that may be made by its manufacturer, is not guaranteed or endorsed by the publisher.
